# Analysis on Metabolic Functions of Stored Rice Microbial Communities by BIOLOG ECO Microplates

**DOI:** 10.3389/fmicb.2018.01375

**Published:** 2018-07-03

**Authors:** Zhiwen Ge, Hengjun Du, Yulong Gao, Weifen Qiu

**Affiliations:** Key Laboratory of Grains and Oils Quality Control and Processing, Collaborative Innovation Center for Modern Grain Circulation and Safety, College of Food Science and Engineering, Nanjing University of Finance and Economics, Nanjing, China

**Keywords:** rice, microbial communities, metabolic functions, BIOLOG ECO microplates, AWCD, principal component analysis

## Abstract

Microbial contamination has been a pervasive issue during the rice storage and triggers extensive researches. The metabolism of microorganisms was proved as an indicator to mirror the degree of microbial contamination. It is necessary to develop a scientific method to analyze the metabolism of rice microbial communities, thereby monitoring the microbial contamination. In this study, the metabolism of rice microbial communities in different storing-year were investigated by BIOLOG ECO microplates. The three rice samples were respectively stored for 1–3 years. The related indicators of BIOLOG ECO microplates were determined, including average well-color development (AWCD) of carbon sources and three metabolic functional diversity indices. The results showed that there were significant differences in the AWCD of all carbon sources among the three rice microbial communities (*p* < 0.05), and the functional diversity indices except Simpson index showed significant differences (*p* < 0.05). Additionally, the three rice microbial communities differed significantly in the metabolic utilization of carboxylic acids and miscellaneous (*p* < 0.05), and there were, however, no significant differences in the other four types of carbon sources. Furthermore, principal component analysis revealed that the microbial communities of stored rice had obviously different metabolic functions in different storage period. Therefore, the study indicated that the BIOLOG ECO microplate was applicable to evaluate the metabolic functions of rice microbial communities, and carboxylic acids and miscellaneous were two crucial parameters of carbon sources to identify the metabolic differences of microbial communities, a case in which it reflected the conditions of rice microbial contamination.

## Introduction

Rice is one of the most important grain crops in the world, and China is the largest rice producer and consumer with yields of approximately 2.09 × 10^11^kg, accounting for about 30% of the world’s production in 2016. Rice is cultivated extensively in China, which is the staple food of Chinese. The storage period of rice is 3 years generally. During rice storage, however, microbial contamination is a common phenomenon, and it directly or indirectly affects the quality and safety of stored rice resulted from the production of mildew and rot as well as pigment secretion (Fleurat-Lessard, 2017). The microbial species and quantities are closely related to the metabolic functions of microbial communities (Amador and Gorres, 2007). Therefore, in order to understand the role of microbial communities in rice storage, it is essential to analyze the metabolic functions of microbial communities (Preston-Mafham et al., 2002), which is meaningful for monitoring and controlling microbial contamination of rice as well.

The plate colony counting method, cell morphology, and RNA/DNA amplification technique were utilized traditionally to evaluate the quantities and structure of microorganisms in microbial communities. These methods, however, definitely had the disadvantages of complex operation protocols, time-consuming assays, low accuracy, and poor repeatability (Jarvis, 2016; Kim et al., 2017). In this case, it is necessary to develop a new method to evaluate the status of microbial communities. The BIOLOG ECO microplate is a detector for the metabolic functions of microbial populations. It contains three sets of system, and each consists of a blank well and 31 different sole carbon source wells, which are closely relevant to the metabolic functions of microbial communities derived from environmental samples (Classen et al., 2003). The absorbance of each well is set as a variable of the sample (Ros et al., 2008; Zhang et al., 2014). The degree of substrate utilization in BIOLOG ECO microplates by microorganisms is measured based on absorbance detected by a redox indicator (Garland, 1996). Microorganisms produce free electrons during the process of using carbon sources, which makes a particular color reaction with the tetrazolium violet, and the depth of the chroma could reflect the carbon source utilization degree of the microorganisms (Agata et al., 2014). Different microorganisms are capable of using different carbon sources, namely the different metabolic functions, a case in which BIOLOG ECO microplate can be potentially applied in the analysis of microbial communities’ status.

The BIOLOG ECO microplate is a relatively simple method, which is commonly applied to characterize the diversity of community-level physiological profiles (Rutgers et al., 2016; Feigl et al., 2017; Jiang et al., 2017). It is a novel method based on the biological and biochemical properties, thereby achieving quick characterization of the ecological status of environmental samples (Agata et al., 2014), such as sediments (Lopes et al., 2016), wastewater (Zhang et al., 2014), activated sludge (Paixão et al., 2007), and soils (Rutgers et al., 2016; Al-Dhabaan and Bakhali, 2017). However, there were little researches analyzing microbial functions of microbial communities by BIOLOG ECO microplates in the area of rice storage.

In this study, the BIOLOG ECO microplate was used to analyze the metabolic functions of rice microbial communities and explore the correlation between storage period and stored rice microbial communities, so as to provide a new method for the safe prevention and control of stored rice.

## Materials and Methods

### Collection of Rice Samples

The rice samples were collected from grain depot of Hunan province, China. Rice was stored at 20 ± 5°C and RH 50 ± 5%, which was a regular grain depot environment. The moisture content of rice was kept under 12%. Each rice sample (150 g) was collected from the center and four corners of three layers of grain depot. Overall, three samples were analyzed. The three rice samples were respectively stored for 1–3 years and correspondingly marked as S1, S2, and S3. The rice was then stored in homogeneous bags at 4°C for the following experiment.

### Preparation of Sample Solution and Plate Cultivation

The rice sample (25 g) was added into 225 ml of 0.85% stroke-physiological saline solution. The mixture was oscillated and shaken for a hour at 4°C, 200 rpm by Controlled Crystal Oscillator (RONGHUA, China), and then stewed for 3 min. The three samples were separately diluted to a 10^-3^ gradient, and each sample was processed in triplicate. Each diluent (150 μl) was added into wells of BIOLOG ECO microplates and the microplates were cultured at a constant temperature (25°C) for 10 days. During cultivation, the ECO microplates were read absorbance values at 590 nm wavelength each 24 h using a Multifunctional Enzyme Label Tester (MOLECULAR DEVICES, United States).

### Determination of Average Well-Color Development Values

Metabolism of the substrate in particular well-results in Formosan production, producing chroma change in the tetrazolium dye (Preston-Mafham et al., 2002). The capability of microorganisms to utilize different carbon sources in microbial communities was measured by average well-color development (AWCD) (Garland and Mills, 1991). Samples with larger variation were thought to have higher carbon source utilization capability and tend to have higher microbial abundance (Garland, 1997). The calculation formula for the AWCD is:

(1) $$\begin{array}{c} \mathrm{AWCD}=\sum_{i=1}^{n}\left(C_{i}-R\right)/n \end{array}$$

In formula (1), C_i_ is the absorbance value of each reaction well at 590 nm, R is the absorbance value of the control well, and n is the number of wells. (C_i_–R) less than 0.06 of wells are calculated as zero (Classen et al., 2003).

### Calculation of Metabolic Functional Diversity Indices

Zak et al. (1994) proposed that the calculation method based on functional diversity indices of BIOLOG ECO microplates could investigate the diversity of communities. Moreover, Keylock (2005) and Strong (2016) extended the concept of evenness to characterize the utilization levels and utilization patterns of microorganisms by carbon source.

(1) Shannon-Wiener diversity index (H′) (Keylock, 2005; Spellerberg, 2008)

(2) $$\begin{array}{c} H^{{}^{\prime }}=-\sum P_{i}\;{\mathrm{lnP}}_{i} \end{array}$$

(3) $$\begin{array}{c} P_{i}=(C_{i}-R)/\sum \left(C_{i}-R\right) \end{array}$$

The P_i_ represents the ratio of the absorbance value in the i^th^ (1 to 31) well to the total absorbance values of all wells.

(2) Shannon evenness index (E) (Keylock, 2005)

(4) $$\begin{array}{c} {E=H}^{{}^{\prime }}/lnS \end{array}$$

S represents the total number of utilized carbon sources (31 carbon sources), the number of wells that vary in color.

(3) Simpson diversity index (D)

(5) $$\begin{array}{c} D=1-\sum P_{i}^{2} \end{array}$$

The above indices reflected the metabolic functional diversity of microbial communities, which was similar to the measurements of diversity indices in general ecology.

### Principal Component Analysis

A BIOLOG ECO microplate contains 31 carbon sources, which are made up with six kinds of carbon sources, including carboxylic acids, carbohydrates, amino acids, polymers, miscellaneous, and amines/amides. In order to avoid the influence on statistical analysis results, we need to convert the absorbance data into R_si_ value to perform principal component analysis (PCA). The calculation formula for the R_si_ is:

(6) $$\begin{array}{c} R_{\mathrm{si}}=(C_{i}-R)/\mathrm{AWCD} \end{array}$$

In formula (6), C_i_ is the absorbance value of each reaction well at different incubation time at 590 nm excluding the control well, and R is the absorbance value of the control well.

In this experiment, measuring the absorbance values of BIOLOG ECO microplates on day 8 were used for the PCA of microbial communities’ metabolic functional diversity.

### Statistical Analysis

All experiments were performed in triplicate, and the results were expressed as means ± standard deviations. Origin 8.5.1 was used to create pictures, and SIMCA 14.1 was used to conduct PCA. Results from one-way analysis of variance (ANOVA) were considered significant when the *p-*value was less than 0.05.

## Results and Discussion

### The AWCD of All Carbon Sources in Rice Microbial Communities Within Incubation Time

**FIGURE 1:**
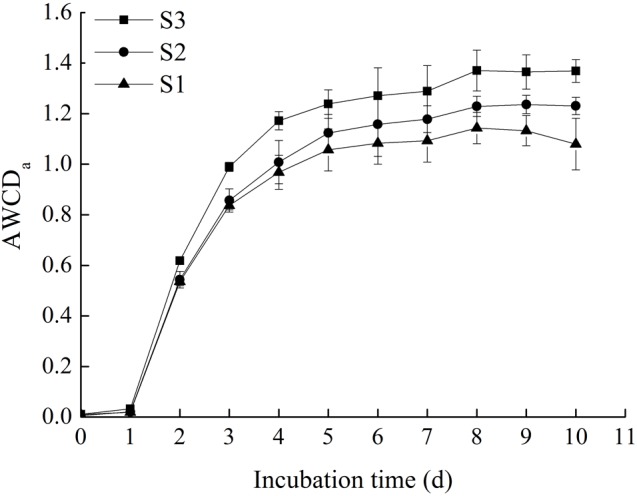
The AWCD of all carbon sources in stored rice microbial communities within incubation time (the original data available in Supplementary Table 1).

Generally, the degree of carbon source oxidation was proportional to the metabolic capability of corresponding microbes, which could be characterized by AWCD (Garland and Mills, 1991). The AWCD_a_ (AWCD of all carbon sources) of the three rice microbial communities are shown in **Figure 1**. The results showed that the AWCD_a_ of all stored rice samples exhibited an apparent lag phase in the first day. Then the average absorbance of all three samples in microplates increased significantly, demonstrating that the three microbial communities were capable of metabolizing organic substrates in BIOLOG ECO microplates. The period selected for metabolic activity analysis was from day 1 to day 8, and the slopes of AWCD_a_ curves within this period represented average metabolic rates of the microbial communities (Kong et al., 2013). During the cultivating period, the increase rate of AWCD_a_ was slower after 5 days. Meanwhile, the AWCD_a_ reached to the peaks on day 8 and the metabolic utilization capability of microbial communities tended to be stable (Zhang et al., 2014), stating that all cultivable microorganisms enable to steadily use carbon sources during the stable period (Miyake et al., 2016). The changes regulation of AWCD_a_ within incubation time was comparable with the studies of Zhang et al. (2014).

Among the three samples, the metabolic rate of S3 was faster than S1 and S2. The AWCD_a_ of S3 increased from 0 to around 1.37 after 8 days. S1 showed the lowest metabolic rate of the substrates in the BIOLOG ECO microplate, and the AWCD_a_ increased to around 1.13 when it got to the stable, which indicated that the utilization of substrates by S1 was less efficient than the others. This illuminated that the storage length of time had an obvious effect on promoting metabolic activity of microorganisms in stored rice. Besides, in the stable period, there were significant differences in the AWCD_a_ among three rice microbial communities (*p* < 0.05), and the order was S3 > S2 > S1, which suggested that the longer storage length of time was, the higher metabolic capability of rice microbial community was. The similar research and method was reported by Sun et al. (2012).

### Metabolism of Different Biochemical Categories of Substrates

According to the biochemical properties of carbon sources, the 31 substrates in the BIOLOG ECO microplates were assigned into six categories, including carboxylic acids, carbohydrates, amino acids, polymers, miscellaneous, and amines/amides (Zhang et al., 2014), the details were described in **Table 2**. The AWCD of different types of carbon sources were classified and analyzed in the experiment.

**FIGURE 2:**
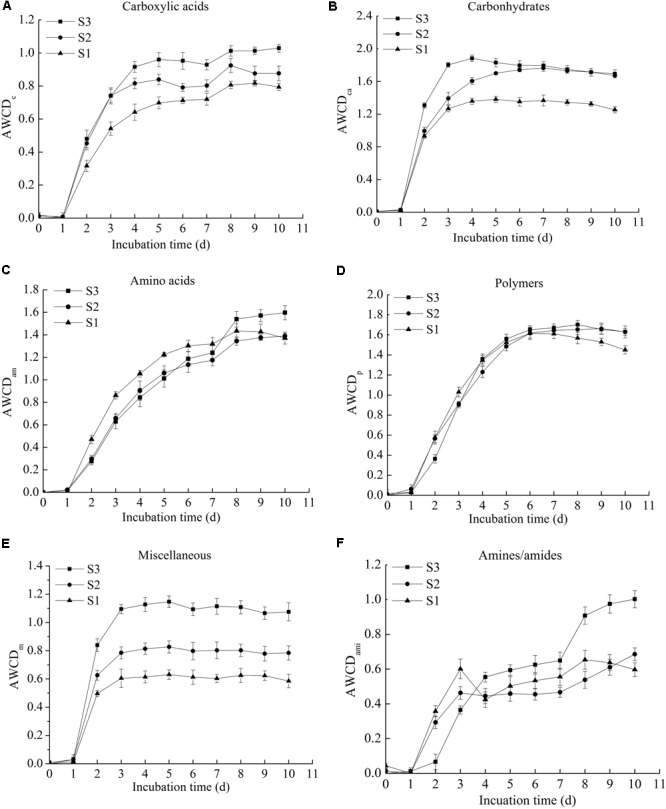
The AWCD of six types of carbon sources in three stored rice microbial communities, including carboxylic acids **(A)**, carbohydrates **(B)**, amino acids **(C)**, polymers **(D)**, miscellaneous **(E)**, and amines/amides **(F)** (the original data available in Supplementary Table 2).

The **Figure 2** showed the AWCD of carboxylic acids, carbohydrates, amino acids, polymers, miscellaneous, and amines/amides. The results indicated that the utilization of six types of carbon sources by microbes presented an increasing trend with the prolongation of incubation time. For carbohydrates, amino acids, polymers and amines/amides, there were no significant difference in the utilization among the three microbial communities. The utilization of carboxylic acids and miscellaneous by the three rice microbial communities, however, differed significantly (*p* < 0.05), and the utilization capability order was S3 > S2 > S1, thereby clarifying that the microbial community of rice increased the utilization of carboxylic acids and miscellaneous with the extendence of storage period.

For different microbial communities, the capability utilizations of six-type carbon sources were different. The AWCD of carbohydrates was the highest, and the lowest was amines/amides, thereby illustrating that carbohydrates were the carbon sources with the highest degree of metabolic utilization, and the lowest degree of metabolic utilization was amines/amides. The results were similar to the findings reported by previous researches, the utilization of carbohydrates was relatively higher than other substrates among the six types of carbon sources, whereas the lowest utilization substrates differed from microbial communities (Kong et al., 2013; Zhang et al., 2014).

In general, through BIOLOG ECO microplates, the metabolic rates of carbon sources were determined by calculating a single value (AWCD) at a single time point, which demonstrated and made a clear comparison of stored rice microbial communities.

### Comparison of Metabolic Functional Diversity Indices

The metabolic functional diversity of microbial communities were actually reflected by functional diversity indices (Zhanga et al., 2013). The Shannon diversity index (H′), Shannon evenness index (E), and Simpson index (D) of rice microbial communities were showed in **Table 1**. Shannon diversity index (H′) is greatly influenced by species richness of communities (Sun et al., 2012). A higher diversity index indicated that the stored rice microbial community metabolic functional diversity was larger (Strong, 2016). The higher the Shannon evenness index (E) was, the more evenly the individuals distributed (Zhanga et al., 2013). Simpson index (D) proposed by Simpson (1949) is greatly reflected by the most common species.

**Table 1:** Comparison of metabolic functional diversity indices of the rice microbial communities.

Sample	Shannon diversity (H′)	Shannon evenness (E)	Simpson diversity (D)
S1	3.378 ± 0.005 a	0.984 ± 0.002 a	0.962 ± 0.001 a
S2	3.313 ± 0.017 c	0.965 ± 0.005 c	0.964 ± 0.002 a
S3	3.340 ± 0.004 b	0.973 ± 0.001 b	0.963 ± 0.001 a

*Data in the table are mean ± SE, *n* = 3. Using Duncan’s multiple range test of diversity indices separately, different letters of each column represent the significant difference at *p* < 0.05.*

Duncan’s multiple range test was separately used in these indices. **Table 1** clearly indicated that two indices except Simpson index (D) of the rice microbial communities had significant difference (*p* < 0.05). The Shannon diversity and evenness index of S1 was highest, followed by sample S3 and S2, indicating that the species richness and evenness of microorganisms in stored rice were relatively high in the early stage of storage. It tended to decrease first and then increased during the storage. Laca et al. (2006) also reported the similar results in their research. They explained that rice carried many field microbes at the beginning of storage, and then field microbes decreased with the extension of storage period, and, meanwhile, the storage microbes increased. However, as shown in **Table 1**, there was no significant difference in Simpson index (D), which manifested that the most common species of the three rice microbial communities were similar, such as fungus of *Aspergillums, Penicillium* and *Mucor*, as observed in previous research (Magan, 2006). Furthermore, the storage length of time has no impact on the common species.

### PCA of Carbon Source Metabolization

Principal component analysis was used to evaluate the carbon source metabolization of microbial communities in a certain environment (Graham and Haynes, 2005; Kong et al., 2013). Some researchers had reported that PCA was applied in the multivariate analysis and enabled to clearly differentiate samples (Felipe-Sotelo et al., 2008; Illian et al., 2009). After dimension reduction, the difference of metabolic characteristics in rice microbial communities were directly reflected by the position of the points in the principal vector space (Graham and Haynes, 2005; Kim et al., 2017).

**FIGURE 3:**
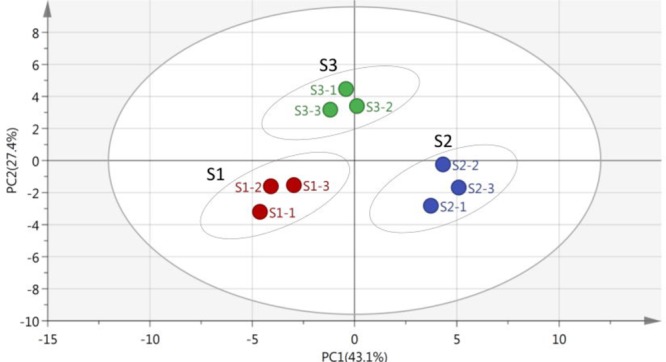
Principal component analysis (PCA) of stored rice microbial communities on the 8th day, the axis of principal component 1 (PC1) described 43.1% and principal component 2 (PC2) described 27.4% of total data variability. The position of the points directly reflected the different metabolic functions of stored rice microbial communities in the principal vector space after dimension reduction (the original data available in Supplementary Table 3).

As shown in **Figure 3**, the multivariate vectors were transformed into two uncorrelated principal component vectors. PC1 presented 43.1% of total variability, and PC2 described 20.42%. For the rice microbial communities, the point position of S1, S2, and S3 were certainly different. As Reddy et al. (2009) stated, the growth and changes of microbial species always occurred during the rice storage period, which led to the difference of capability utilization of microbial communities.

**Table 2** showed the loading scores of 31 carbon sources in the first two principal components. The higher loading scores were, the larger effects of carbon source on the principal components were. The sample distribution in the PC axis was relevant to carbon source substrates capability utilization of stored rice microbes (Zhanga et al., 2013). From the PCA results in **Table 2**, it could be seen that there were 17 kinds of carbon sources had mainly impacts on the PC1. The 17 kinds of carbon sources included five kinds of carbohydrates, four kinds of amino acid, three kinds of carboxylic acid, two kinds of miscellaneous, polymers and amines/amides. These results indicated that the carbon sources with impacts on the PC1 were mainly carbohydrates, amino acid, and miscellaneous. Furthermore, there were mainly 14 types of carbon sources with effects on the PC2, including six kinds of carboxylic acids, three kinds of carbohydrates, two kinds of amino acids and polymers, a kind of miscellaneous. Thereby, the carboxylic acids made the major effects on the PC2. From the results of carbon sources analysis above, it was obvious that the carbon sources utilized by rice microbes were carbohydrates, amino acid, miscellaneous, and carboxylic acids. The similar analysis method was reported by Zhanga et al. (2013).

**Table 2:** The 31 kinds of carbon substrates loaded on the first and second principal component in analysis of BIOLOG ECO microplates data.

Chemical guild	Plate number	Substrates	Chemical formula	PC1	PC2
Miscellaneous	B1	Pyruvic acid methyl ester	C_4_H_6_O_3_	0.2133	0.1968
	G2	Glucose-1-phosphate	C_6_H_13_O_9_P	0.0033	0.3322
	H2	D,L-α-Glycerol phosphate	C_3_H_9_O_6_P	–0.2420	0.1032
Polymers	C1	Tween 40	–	0.2398	–0.0996
	D1	Tween 80	–	0.0500	–0.1901
	E1	α-Cyclodextrin	C_36_H_60_O_30_	0.1103	–0.1781
	F1	Glycogen	(C_6_H_10_O_5_)_n_	0.2141	0.0407
Carbohydrates	G1	D-Cellobiose	C_12_H_12_O_11_	0.2292	0.0885
	H1	α-D-Lactose	C_12_H_12_O_11_	0.2426	0.1402
	A2	Methyl-D-glucoside	C_7_H_14_O_6_	0.2489	0.0717
	B2	D-Xylose	C_5_H_10_O_5_	0.1942	–0.1073
	C2	i-Erythritol	C_4_H_10_O_4_	0.2296	–0.0517
	D2	D-Mannitol	C_6_H_14_O_6_	0.1226	–0.1551
	E2	*N*-Acetyl-D-glucosamine	C_8_H_15_NO_6_	0.0929	0.2089
Carboxylic acids	F2	D-Glucosaminic acid	C_6_H_13_NO_6_	0.1849	0.1302
	A3	D-Galactonic acid latone	C_6_H_10_O_6_	0.2058	0.1997
	B3	D-Galacturonic acid	C_6_H_10_O_7_	0.1217	0.1913
	C3	2-Hydroxy benzoic acid	C_7_H_6_O_3_	–0.1194	–0.2752
	D3	4-Hydroxy benzoic acid	C_7_H_6_O_3_	0.0291	0.3345
	E3	γ-Hydroxy butyric acid	C_4_H_8_O_3_	–0.1384	0.2710
	F3	Itaconic acid	C_5_H_6_O_4_	0.1027	–0.1428
	G3	α-Keto butyric acid	C_4_H_6_O_3_	0.0846	0.2812
	H3	D-Malic acid	C_4_H_6_O_5_	0.1845	0.1137
Amino acids	A4	L-Arginine	C_4_H_14_N_4_O_2_	–0.2298	0.1084
	B4	L-Asparagine	C_4_H_8_N_2_O_3_	0.1433	–0.0043
	C4	L-Phenylalanine	C_9_H_11_NO_2_	–0.0231	–0.2425
	D4	L-Serine	C_3_H_7_NO_3_	0.2494	0.0564
	E4	L-Threonine	C_4_H_9_NO_3_	–0.2045	0.1562
	F4	Glycyl-L-glutamic acid	C_7_H_12_N_2_O_5_	0.0982	–0.2394
Amines/amides	G4	Phenylethylamine	C_8_H_11_N	–0.2682	–0.0264
	H4	Putrescine	C_4_H_12_N_2_	–0.2316	0.1527

## Conclusion

In present study, the metabolic functions of microbial communities of rice with different storage years were investigated and analyzed by BIOLOG ECO microplates. With the extension of storage period, the utilization of rice microbial communities for carboxylic acids and miscellaneous increased. Among the six types of carbon sources, the utilization of carbohydrates was relatively higher than other substrates. The metabolic functional diversity indices showed that there were differences among the three rice microbial communities, whereas the most common species of them were similar. Furthermore, carbon sources utilization of the three microbial communities were certainly different, and the carbon sources mainly utilized by stored rice microbes were carbohydrates, amino acid, miscellaneous, and carboxylic acids.

Therefore, to draw a conclusion, this study illustrated that BIOLOG ECO microplates were capable of preceding a quick and accurate determination of metabolic functions of rice microbial communities, which provided a novel method for safe prevention and control of stored rice. However, BIOLOG ECO microplates are unable to evaluate the microbial communities’ structure of stored rice. The evaluation of dominant species as well as species distribution and difference of three stored rice microbial communities need to be studied in the next work.

## Author Contributions

ZG: conceived and designed the experiments, performed the experiments, data analyses and wrote the manuscript, and contributed the experimental materials. ZG, HD, and YG: revised the manuscript, played an important role in interpreting the results, and helped perform the analysis with constructive discussions. WQ: approved the final version.

## Conflict of Interest Statement

The authors declare that the research was conducted in the absence of any commercial or financial relationships that could be construed as a potential conflict of interest.
