# Supplementary material for: Analysis on Metabolic Functions of Stored Rice Microbial Communities by BIOLOG ECO Microplates
Source: Front Microbiol. 2018 Jul 3;9:1375. doi: 10.3389/fmicb.2018.01375 (PMC6037723; doi:10.3389/fmicb.2018.01375)
Supplement: Supplementary file 1 [file Data_Sheet_1.docx]

**Supplementary Material:**

**Supplementary Table 1.** **The original data of Figure 1**

|  | **Changes of AWCD in stored rice microbial communities** | | | | | |
| --- | --- | --- | --- | --- | --- | --- |
| **Incubation time /d** | **2014** | **STDEV** | **2015** | **STDEV** | **2016** | **STDEV** |
| 0 | 0.0113 | 0.0022 | 0.0053 | 0.0001 | 0.0097 | 0.0008 |
| 1 | 0.0325 | 0.0053 | 0.0205 | 0.0128 | 0.0191 | 0.0036 |
| 2 | 0.6182 | 0.0114 | 0.5438 | 0.03289 | 0.5354 | 0.01556 |
| 3 | 0.9896 | 0.01795 | 0.8567 | 0.04606 | 0.8367 | 0.01736 |
| 4 | 1.1717 | 0.03603 | 1.0084 | 0.0855 | 0.9683 | 0.06727 |
| 5 | 1.2382 | 0.0559 | 1.1237 | 0.07363 | 1.0567 | 0.08299 |
| 6 | 1.2713 | 0.1101 | 1.1578 | 0.12707 | 1.0833 | 0.0827 |
| 7 | 1.2891 | 0.1016 | 1.1783 | 0.05285 | 1.0927 | 0.08409 |
| 8 | 1.3708 | 0.0807 | 1.2288 | 0.03994 | 1.1432 | 0.06164 |
| 9 | 1.3648 | 0.0682 | 1.2359 | 0.03636 | 1.1327 | 0.0597 |
| 10 | 1.3688 | 0.0453 | 1.2305 | 0.03439 | 1.0799 | 0.1021 |

**Supplementary Table 2.** **The original data of Figure 2(A-F)**

| **Carboxylic acids (Figure 2A)** | | | | | | |
| --- | --- | --- | --- | --- | --- | --- |
| **Incubation time/d** | **2014** | **STDEV** | **2015** | **STDEV** | **2016** | **STDEV** |
| 0 | 0 | 0 | 0.0135 | 0.0168 | 0.0184 | 0.0164 |
| 1 | 0.0063 | 0.0056 | 0.0074 | 0.0248 | 0.0029 | 0.0057 |
| 2 | 0.4797 | 0.0536 | 0.4522 | 0.039 | 0.3165 | 0.0342 |
| 3 | 0.7405 | 0.0487 | 0.7417 | 0.0388 | 0.5421 | 0.0402 |
| 4 | 0.9156 | 0.0329 | 0.8156 | 0.0317 | 0.6419 | 0.0494 |
| 5 | 0.9598 | 0.0425 | 0.8394 | 0.0319 | 0.6981 | 0.0355 |
| 6 | 0.9521 | 0.0511 | 0.7909 | 0.0248 | 0.7128 | 0.0194 |
| 7 | 0.9285 | 0.0307 | 0.8021 | 0.035 | 0.7196 | 0.0358 |
| 8 | 1.0124 | 0.0313 | 0.9245 | 0.0419 | 0.8062 | 0.0221 |
| 9 | 1.0131 | 0.0217 | 0.876 | 0.0448 | 0.8167 | 0.0169 |
| 10 | 1.029 | 0.0233 | 0.8763 | 0.045 | 0.7937 | 0.021 |

| **Carbonhydrates (Figure 2B)** | | | | | | |
| --- | --- | --- | --- | --- | --- | --- |
| **Incubation time/d** | **2014** | **STDEV** | **2015** | **STDEV** | **2016** | **STDEV** |
| 0 | 0.0102 | 0.0004 | 0.0099 | 0.0057 | 0.0095 | 0.0009 |
| 1 | 0.0232 | 0.0022 | 0.0291 | 0.0189 | 0.0247 | 0.0021 |
| 2 | 1.3075 | 0.0315 | 0.9942 | 0.0461 | 0.931 | 0.0266 |
| 3 | 1.8024 | 0.028 | 1.3926 | 0.0749 | 1.2674 | 0.0383 |
| 4 | 1.8826 | 0.0404 | 1.6056 | 0.0412 | 1.3585 | 0.0385 |
| 5 | 1.8302 | 0.0541 | 1.6996 | 0.0063 | 1.381 | 0.0387 |
| 6 | 1.7966 | 0.0567 | 1.7424 | 0.0251 | 1.3521 | 0.052 |
| 7 | 1.795 | 0.0462 | 1.7636 | 0.0389 | 1.3664 | 0.0658 |
| 8 | 1.7482 | 0.0483 | 1.7302 | 0.0155 | 1.3453 | 0.0354 |
| 9 | 1.7137 | 0.0464 | 1.7142 | 0.0115 | 1.325 | 0.0287 |
| 10 | 1.6931 | 0.0502 | 1.6715 | 0.0144 | 1.2531 | 0.0382 |

| **Amino acids (Figure 2C)** | | | | | | |
| --- | --- | --- | --- | --- | --- | --- |
| **Incubation time/d** | **2014** | **STDEV** | **2015** | **STDEV** | **2016** | **STDEV** |
| 0 | 0 | 0 | 0 | 0 | 0 | 0 |
| 1 | 0.0144 | 0.0077 | 0.0225 | 0.0135 | 0 | 0 |
| 2 | 0.2769 | 0.0326 | 0.2974 | 0.031 | 0.4707 | 0.0373 |
| 3 | 0.6285 | 0.0625 | 0.6584 | 0.0445 | 0.8623 | 0.0325 |
| 4 | 0.8435 | 0.082 | 0.9039 | 0.0861 | 1.0543 | 0.0295 |
| 5 | 1.012 | 0.0757 | 1.0602 | 0.0641 | 1.2243 | 0.0189 |
| 6 | 1.1874 | 0.0618 | 1.1348 | 0.068 | 1.3032 | 0.0489 |
| 7 | 1.2407 | 0.064 | 1.1744 | 0.04751 | 1.3186 | 0.0607 |
| 8 | 1.539 | 0.0698 | 1.3445 | 0.0363 | 1.4345 | 0.0629 |
| 9 | 1.5714 | 0.0529 | 1.3746 | 0.0248 | 1.4262 | 0.0698 |
| 10 | 1.5973 | 0.0624 | 1.3888 | 0.0261 | 1.371 | 0.0529 |

| **Polymers (Figure 2D)** | | | | | | |
| --- | --- | --- | --- | --- | --- | --- |
| **Incubation time/d** | **2014** | **STDEV** | **2015** | **STDEV** | **2016** | **STDEV** |
| 0 | 0 | 0 | 0 | 0 | 0.0174 | 0.037 |
| 1 | 0.0286 | 0.0131 | 0.0624 | 0.0426 | 0.0296 | 0.0293 |
| 2 | 0.3637 | 0.0423 | 0.566 | 0.0379 | 0.5761 | 0.065 |
| 3 | 0.9031 | 0.0313 | 0.9126 | 0.022 | 1.0299 | 0.0481 |
| 4 | 1.3606 | 0.0479 | 1.2291 | 0.055 | 1.3464 | 0.0497 |
| 5 | 1.5585 | 0.0476 | 1.4849 | 0.0429 | 1.5245 | 0.0545 |
| 6 | 1.6524 | 0.0385 | 1.6168 | 0.0504 | 1.6153 | 0.061 |
| 7 | 1.6707 | 0.0387 | 1.644 | 0.0415 | 1.6105 | 0.0483 |
| 8 | 1.7015 | 0.042 | 1.6538 | 0.052 | 1.5685 | 0.0564 |
| 9 | 1.655 | 0.0461 | 1.6623 | 0.0561 | 1.5299 | 0.0367 |
| 10 | 1.6304 | 0.0336 | 1.6293 | 0.0587 | 1.4502 | 0.0408 |

| **Miscellaneous (Figure 2E)** | | | | | | |
| --- | --- | --- | --- | --- | --- | --- |
| **Incubation time/d** | **2014** | **STDEV** | **2015** | **STDEV** | **2016** | **STDEV** |
| 0 | 0 | 0 | 0.0088 | 0.0153 | 0 | 0 |
| 1 | 0.0297 | 0.0129 | 0.0295 | 0.0341 | 0.0148 | 0.0314 |
| 2 | 0.8395 | 0.0446 | 0.6262 | 0.0347 | 0.4962 | 0.0221 |
| 3 | 1.0949 | 0.0328 | 0.7849 | 0.0428 | 0.605 | 0.0643 |
| 4 | 1.1273 | 0.0497 | 0.8138 | 0.0404 | 0.6141 | 0.0419 |
| 5 | 1.1469 | 0.0406 | 0.8266 | 0.0431 | 0.6311 | 0.0337 |
| 6 | 1.0928 | 0.0445 | 0.7975 | 0.0624 | 0.6129 | 0.0502 |
| 7 | 1.1142 | 0.0568 | 0.8024 | 0.0598 | 0.6036 | 0.0283 |
| 8 | 1.1077 | 0.0468 | 0.8023 | 0.0451 | 0.6252 | 0.0508 |
| 9 | 1.0654 | 0.0442 | 0.7787 | 0.0526 | 0.6234 | 0.0339 |
| 10 | 1.0753 | 0.0641 | 0.7846 | 0.05 | 0.5855 | 0.0476 |

| **Amines/amides (Figure 2F)** | | | | | | |
| --- | --- | --- | --- | --- | --- | --- |
| **Incubation time/d** | **2014** | **STDEV** | **2015** | **STDEV** | **2016** | **STDEV** |
| 0 | 0 | 0 | 0.0146 | 0.0252 | 0.0438 | 0.0156 |
| 1 | 0.0115 | 0.0199 | 0 | 0 | 0 | 0 |
| 2 | 0.0664 | 0.0443 | 0.2932 | 0.0356 | 0.3576 | 0.0324 |
| 3 | 0.3649 | 0.0237 | 0.4629 | 0.0359 | 0.6008 | 0.0562 |
| 4 | 0.554 | 0.0284 | 0.4451 | 0.0439 | 0.4243 | 0.0447 |
| 5 | 0.5939 | 0.031 | 0.459 | 0.0442 | 0.503 | 0.053 |
| 6 | 0.6253 | 0.0532 | 0.4547 | 0.0322 | 0.534 | 0.0456 |
| 7 | 0.6494 | 0.0492 | 0.4665 | 0.029 | 0.5559 | 0.0493 |
| 8 | 0.9079 | 0.0495 | 0.538 | 0.0484 | 0.6535 | 0.0541 |
| 9 | 0.975 | 0.0529 | 0.6108 | 0.046 | 0.6376 | 0.0462 |
| 10 | 1.0024 | 0.0497 | 0.6859 | 0.036 | 0.5968 | 0.0395 |

**Supplementary Table 3.** **The original data of Figure 3**

| **The Rsi values of 2014-year stored rice on the 8^th^ day** | | | | |
| --- | --- | --- | --- | --- |
|  | **1** | **2** | **3** | **4** |
| **A** |  | 1.2933 | 0.8652 | 1.3113 |
| **B** | 1.1990 | 1.1114 | 1.3418 | 1.5468 |
| **C** | 1.3880 | 1.3717 | -0.0927 | 0.6739 |
| **D** | 1.4273 | 1.4302 | 0.9138 | 1.3587 |
| **E** | 1.3506 | 1.3138 | 0.1293 | 1.4475 |
| **F** | -0.0098 | 1.3671 | 0.9613 | 1.2138 |
| **G** | 1.0825 | 0.9120 | 0.2724 | 0.4761 |
| **H** | 1.3084 | -0.0556 | 1.1572 | 0.9338 |
|  | **5** | **6** | **7** | **8** |
| **A** |  | 1.2227 | 0.5520 | 1.4262 |
| **B** | 1.3891 | 1.1387 | 1.1015 | 1.2461 |
| **C** | 1.3427 | 1.3840 | -0.0553 | 1.1925 |
| **D** | 1.3047 | 1.2876 | 0.7987 | 1.3298 |
| **E** | 1.3398 | 1.3421 | 1.3752 | 0.8543 |
| **F** | 1.3422 | 1.3323 | -0.0377 | 1.3425 |
| **G** | 1.2340 | 0.9248 | 0.5106 | 0.3403 |
| **H** | 1.1637 | -0.0287 | 0.8500 | 0.4537 |
|  | **9** | **10** | **11** | **12** |
| **A** |  | 1.1456 | 1.1425 | 0.4955 |
| **B** | 1.3865 | 1.1854 | 1.1775 | 0.8602 |
| **C** | 1.4867 | 1.4784 | -0.0156 | 0.4702 |
| **D** | 1.3620 | 1.2647 | 0.3989 | 1.2862 |
| **E** | 1.1506 | 1.4348 | 1.4396 | 1.3549 |
| **F** | 1.3744 | 1.2731 | 0.0145 | 0.8726 |
| **G** | 1.3797 | 1.1772 | 0.2977 | 0.7019 |
| **H** | 1.2961 | 0.2210 | 0.7720 | 1.1152 |

| **The Rsi values of 2015-year stored rice on the 8^th^ day** | | | | |
| --- | --- | --- | --- | --- |
|  | **1** | **2** | **3** | **4** |
| A |  | 1.3992 | 0.5136 | 0.7975 |
| B | 0.8784 | 1.1560 | 1.0316 | 1.5913 |
| C | 1.6856 | 1.6138 | 0.0580 | 1.4448 |
| D | 1.3627 | 1.4343 | 0.2639 | 1.0596 |
| E | 1.5125 | 1.1363 | 0.8469 | 0.9798 |
| F | 0.3111 | 1.5720 | 0.9031 | 1.4356 |
| G | 1.3084 | 0.6476 | 0.9796 | 0.1520 |
| H | 1.2967 | 0.1002 | 1.0727 | 0.4555 |
|  | **5** | **6** | **7** | **8** |
| A |  | 1.3211 | 1.0638 | 1.1929 |
| B | 0.9449 | 1.6369 | 1.3638 | 1.5027 |
| C | 1.3624 | 1.5274 | -0.0866 | 1.3182 |
| D | 1.4808 | 1.4106 | 0.4208 | 1.4225 |
| E | 1.0619 | 1.3830 | -0.0088 | 0.6417 |
| F | 1.5277 | 1.1748 | -0.1512 | 1.3805 |
| G | 1.5650 | 0.8832 | 0.5382 | 0.3331 |
| H | 1.4761 | -0.0674 | 0.9293 | 0.4509 |
|  | **9** | **10** | **11** | **12** |
| A |  | 1.3673 | 0.3513 | 0.7866 |
| B | 1.4292 | 1.3290 | 0.8612 | 1.1733 |
| C | 1.7321 | 1.6367 | 0.0613 | 0.9024 |
| D | 1.6368 | 1.4998 | 0.3252 | 1.5026 |
| E | 1.3735 | 1.3862 | 0.7011 | 0.1954 |
| F | 1.5175 | 1.0952 | 1.6529 | 0.4417 |
| G | 1.3807 | 0.6963 | 0.1779 | 0.0602 |
| H | 1.4154 | 0.1093 | 1.0171 | 1.1848 |
|  |  |  |  |  |

| **The Rsi values of 2016-year stored rice on the 8^th^ day** | | | | |
| --- | --- | --- | --- | --- |
|  | **1** | **2** | **3** | **4** |
| A |  | 0.8618 | 0.5146 | 1.3636 |
| B | 0.6641 | 1.0342 | 0.7768 | 1.4936 |
| C | 1.3940 | 1.3822 | 0.1440 | 1.5715 |
| D | 1.5159 | 1.1503 | 0.1669 | 1.1279 |
| E | 1.3162 | 1.2365 | 1.1236 | 1.3278 |
| F | 1.2336 | 1.0717 | 0.8561 | 1.3374 |
| G | 1.0289 | 0.6991 | 0.5444 | 1.2488 |
| H | 0.8131 | 0.2386 | 0.7626 | 1.0002 |
|  | **5** | **6** | **7** | **8** |
| A |  | 0.9123 | 0.5874 | 1.4984 |
| B | 0.9392 | 1.1160 | 1.1280 | 1.4263 |
| C | 1.2754 | 1.3710 | -0.1554 | 1.2200 |
| D | 1.4425 | 1.2112 | 0.0900 | 1.2591 |
| E | 1.3997 | 1.2932 | 0.9856 | 1.2074 |
| F | 1.3023 | 1.0247 | 1.2754 | 1.3585 |
| G | 1.1019 | 0.6441 | 0.5373 | 1.0740 |
| H | 0.9082 | -0.0336 | 0.8169 | 0.7830 |
|  | **9** | **10** | **11** | **12** |
| A |  | 1.1682 | 0.3941 | 1.2673 |
| B | 0.8706 | 1.3543 | 0.9195 | 1.1379 |
| C | 1.5103 | 1.6216 | 0.0484 | 1.3305 |
| D | 1.5055 | 1.6374 | 0.3438 | 1.2178 |
| E | 1.2615 | 1.3369 | 0.9811 | 0.6402 |
| F | 1.3441 | 1.1878 | 1.0361 | 0.7316 |
| G | 1.2091 | 0.8656 | 0.8026 | 0.1393 |
| H | 1.1084 | 0.1527 | 1.0354 | 0.8404 |
